# T peripheral helper cells in autoimmune diseases: What do we know?

**DOI:** 10.3389/fimmu.2023.1145573

**Published:** 2023-04-03

**Authors:** Yao Huang, Xin Ba, Liang Han, Hui Wang, Weiji Lin, Zhe Chen, Shenghao Tu

**Affiliations:** ^1^ Department of Integrated Traditional Chinese and Western Medicine, Tongji Hospital, Tongji Medcal College, Huazhong University of Science and Technology, Wuhan, China; ^2^ Institute of Integrated Traditional Chinese and Western Medicine, Tongji Hospital, Tongji Medical College, Huazhong University of Science and Technology, Wuhan, China; ^3^ Rehabilitation & Sports Medicine Research Institute of Zhejiang, Zhejiang Provincial People’s Hospital, People’s Hospital of Hangzhou Medical College, Hangzhou, China

**Keywords:** T peripheral helper cells, autoimmune diseases, CXCL13, tertiary lymphoid structures (TLSs), T follicular helper cells

## Abstract

The interactions between T cells and B cells are essential for antibody responses and the development of autoimmune diseases. Recently, a distinct subset of T cells capable of helping B cells was established in synovial fluid, and they were termed peripheral helper T (Tph) cells. PD-1^hi^CXCR5^−^CD4^+^ Tph cells express high levels of CXCL13, which drives the formation of lymphoid aggregates and tertiary lymphoid structures, ultimately facilitating the local production of pathogenic autoantibodies. Tph and T follicular helper cells share some key features but can be distinguished by their surface markers, transcriptional regulation, and migration capability. We summarize recent findings on Tph cells in this review and provide a perspective on their potential roles in a range of autoimmune diseases. More clinical and in-depth mechanistic investigations of Tph cells may help to improve the understanding of pathogenesis and further provide novel therapeutic targets in autoimmune diseases.

## 1 Introduction

Autoimmune diseases are characterized by the breakdown of immune tolerance, recognition of self-​antigens, and hyperactivity of adaptive immune responses, leading to the production of specific autoantibodies and ultimately attack multiple organs of the body (1). The pathogenesis and development of autoimmune diseases is largely dependent on immune responses, which are mediated by interactions between T cells and B cells. With the advancement of experimental technology and in-depth research, it was recognized that follicular helper T (Tfh) and follicular regulatory T (Tfr) cells play significant roles in the production of high‐affinity antibodies in germinal centers (GCs). These CXCR5^+^ T cells are directed to B‐cell follicles by gradients of CXCL13 and orchestrate the GC responses (2, 3).

Recently, a distinct subset of helper T cells was identified in the synovial fluid (SF) T cells from patients with rheumatoid arthritis (RA). These cells are characterized by the absence of the Tfh-cell markers CXCR5 and Bcl6, and the expression of high levels of CXCL13 (4). CXCL13 is crucial for the recruitment of B and T cells and the formation of lymphoid structures (5). Subsequently, another study conducted in RA found that CXCL13-producing CD4^+^ T cells could recruit CXCR5^+^ cells, such as Tfh and B cells. These CD4^+^PD-1^+^CXCR5^−^cells were lack of IFN-γ, IL-4, IL-17, and Foxp3, and they had lower expression of ICOS compared with Tfh cells (6). Until 2017, the PD-1^hi^CXCR5^−^ T cell subpopulation was defined as a distinct T cell subset based on multidimensional cytometry, transcriptomics, and functional assays (7). This population was significantly expanded in the synovial tissue from seropositive RA patients and constituted about 85% of synovial PD-1^hi^CD4^+^ cells. Due to their capacity of B cell help, this population of cells was termed peripheral helper T (Tph) cells (7). Tph and Tfh cells were found adjacent to B cells within lymphoid aggregates. However, more Tph cells than Tfh cells were found adjacent to B cells in areas outside of lymphoid aggregates (7). Similar to Tfh cells, Tph cells could induce plasma cell differentiation *via* interactions between IL-21 and SLAMF5. On the other hand, the expression of Bcl6, Blimp-1, and chemokine receptors including CCR2, CX3CR1, and CCR5 distinguished Tph cells from Tfh cells (7). Accumulating studies have shown that Tph cells are involved in a range of diseases, such as autoimmune diseases and malignancies (8, 9). Here, we review recent studies on Tph cells and their roles in autoimmune diseases, which may provide novel disease biomarker and therapeutic strategies for autoimmune diseases.

## 2 Tph and tertiary lymphoid structures (TLSs)

**Figure 1:**
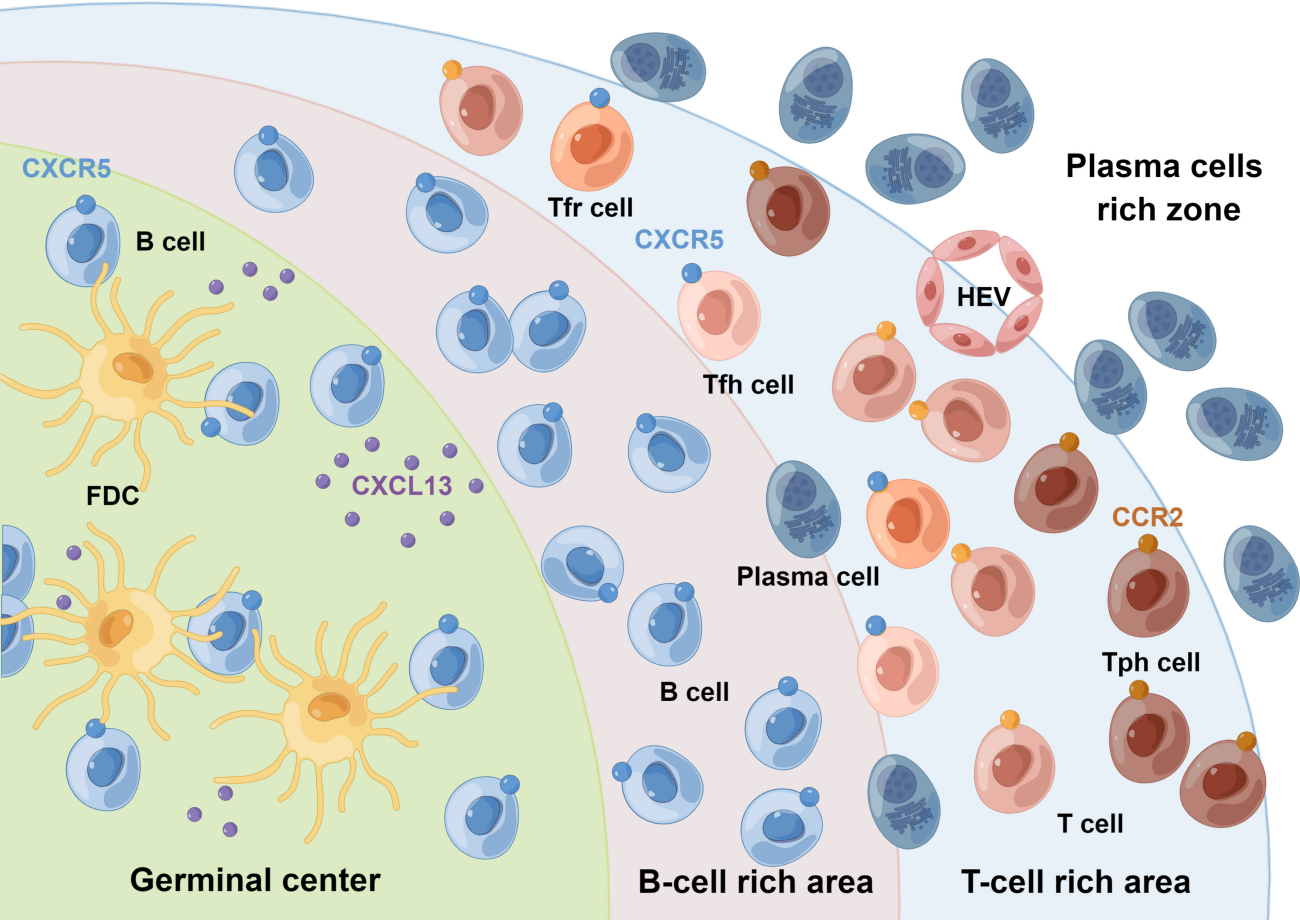
Schematic representation of synovial TLSs. Synovial TLSs show striking anatomical similarities to SLOs. Within the lympho-myeloid group of RA patients, TLSs resemble the lymphoid follicles of SLOs, acquiring features such as segregation of T and B cells into separate areas, differentiation of the FDCs networks, development of HEVs, and differentiation of hypermutated and class-switched plasma cells. Tph cells are supposed to be present in the periphery of TLSs, rather than in their center. These crosstalks among Tph, Tfh, Tfr, and B cells regulate the GC response. FDC, follicular dendritic cell; HEV, high endothelial venule. By Figdraw.

Lymphoid tissues can be broadly classified into primary and secondary lymphoid organs (SLOs). In multiple chronic inflamed tissues, stromal cells acquire the properties of SLOs and drive the formation of ectopic clusters of lymphomonocytic cells, named TLSs, also known as ectopic lymphoid structures (10). TLSs recapitulate the cellular, molecular, and structural organization of SLOs, comprising follicular dendritic cells (FDCs), fibroblastic reticular cells, antigen-presenting cells, lymphatic sinuses, and high endothelial venules (HEVs), but lack the surrounding capsule in most tissues (11). This structure may allow their cellular components to enter the surrounding tissue directly, facilitating the generation or enhancement of adaptive immune responses (12). They are mostly located in organs or tissues that are not predisposed to allow the formation of lymphoid tissues during embryonic development, such as synovium (13), salivary glands (14), meninges, kidneys, etc (15). The presence of TLSs is regarded as a unique feature shared by various chronic inflammatory diseases, including autoimmune diseases, as well as the tumor immune microenvironment (11, 12). Researches conducted in RA found that TLSs were predominantly located within the sublining of synovial tissues and were present in some patients with early untreated RA (16). The prevalence of TLSs in RA varies widely depending on the site of biopsy, treatment strategy, and stage of disease (17–21). Within the lympho-myeloid group of RA patients, TLSs acquired several SLO-like features (16), such as segregation of T and B cells into separate areas, differentiation of the FDCs networks, development of HEVs, and differentiation of hypermutated and class-switched plasma cells (**Figure 1**). TLSs are enriched in pro-inflammatory cytokines but lack key checkpoints for autoreactive cell screening, suggesting that they may be critical for the local production of pathogenic autoantibodies (22–24).

A functional model of Tph cells in peripheral tissues has been proposed, in which Tph cells infiltrate peripheral tissues in the context of persistent chronic inflammation (25). Locally activated Tph cells produce very high levels of CXCL13, a chemokine that binds to CXCR5 and leads to the recruitment of B and Tfh cells and subsequent formation of lymphoid follicles (5). Similar to Tfh cells, Tph cells produce IL-21, which induces B cell activation and differentiation into plasmablasts (7, 26). Tfr cells are deemed as repressors of GC reactions, which possess dual characteristics of Tfh cells and traditional Treg cells (2, 27). Interestingly, Tfr cells have also been found in tumor-infiltrating lymphocytes and are associated with TLS activities (28). These crosstalks among Tph, Tfh, Tfr, and B cells may allow the formation of more mature structures that eventually form TLSs, which then facilitate the selection of antigen-driven B cell clones through affinity maturation and further promote adaptive immune responses in situ.

TLSs have been identified in target tissues from patients with RA (13), Sjögren syndrome (SS) (14), systemic lupus erythematosus (SLE) (29), and myositis (30, 31). TLSs in autoimmune diseases not only bear striking anatomical resemblance to SLOs, but can also show features of functional GCs. B cells within TLSs express the cytidine deaminase AID (17), the enzyme which is important for immunoglobulin somatic cell hypermutation and class switching (32). Evidence suggests that TLSs contribute to the perpetuation of local autoimmunity to disease-associated autoantigens such as anti-citrullinated protein antibodies (17, 33). Furthermore, they appear to be associated with serum autoantibody concentrations, disease severity and progression, organ function, and response to therapy (34). However, the exact mechanism of Tph cells in the formation and maintenance of TLSs as well as their contribution to disease pathogenesis still remain to be elucidated.

Thus, in conclusion, Tph cells contribute to the formation of lymphoid aggregates and TLSs, ultimately facilitating the local production of pathogenic autoantibodies directly in inflamed tissues. Notably, Tph cells are not hardwired to the presence of TLSs. Instead, they can be found in patients without TLSs (35) or even in chronic inflammatory diseases that do not develop TLSs at all (36).

## 3 Similarities and differences between Tph and Tfh cells

**Figure 2:**
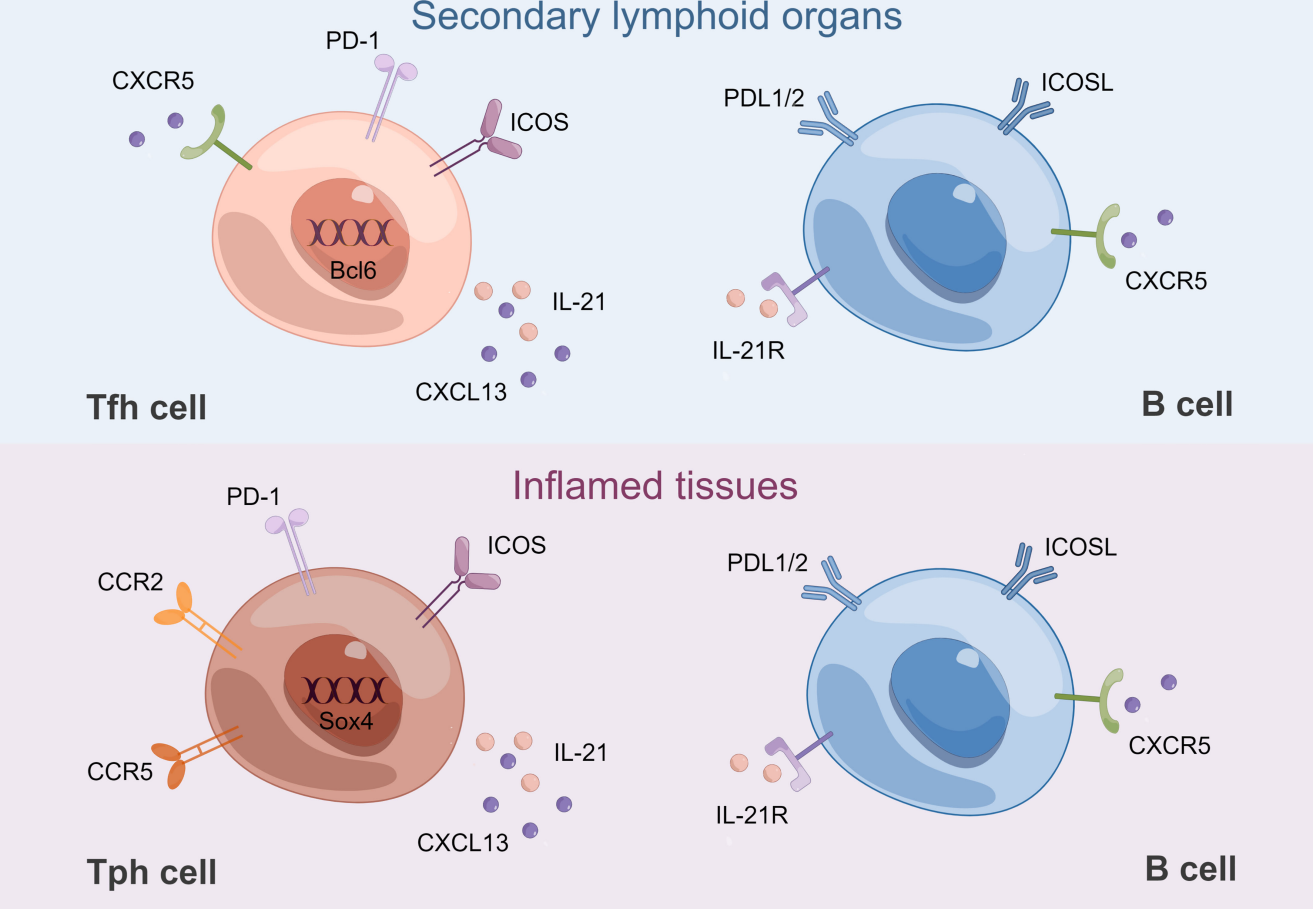
B-Tfh cell and B-Tph cell crosstalk. Tfh cells and B cells interact in GCs of SLOs. Bcl6 is a lineage-defining transcription factor of Tfh cells. Tph cells and B cells interact in inflamed tissues. Sox4 is a key transcription factor for CXCL13 production by Tph cells. CCR2 and CCR5 contribute to their recruitment to inflamed tissues. Both Tfh and Tph cells express high levels of IL-21 and CXCL13, which are mandatory for B-cell differentiation. Both Tfh and Tph cells interact with B cells through their respective expression of ICOS, PD-1 and ICOSL, PDL1/2. ICOSL, ICOS ligand; PDL1/2: programmed death-ligand1/2. By Figdraw.

Since the last decade, Tfh cells have been identified as a specialized subset of CD4^+^ T cells capable of providing help to B cells in GCs. Bcl6 was discovered as a lineage-defining transcription factor of Tfh cells (3, 37, 38). As B-cell helper cells, PD-1^hi^CXCR5^−^ Tph and PD-1^hi^CXCR5^+^Tfh cells share some key features (**Figure 2**). Both subsets express IL-21, CXCL13, and ICOS, thus possessing B cell helper function (7). Transcriptomic analyses revealed a strong overlap of gene signature between Tfh and Tph cells, including MAF, TIGIT, and SLAMF6 (7). Compared with those in PD-1^−^CXCR5^−^ cells, the expression of 11 proteins, including TIGIT, ICOS, CD38, and CD57 was significantly increased, while the expression of 5 proteins, including CD25 and CD127, was significantly decreased in Tfh and Tph cells (7). Notably, they both induce plasma cell differentiation through the interactions between IL-21 and SLAMF5 (7, 39). The differentiation mechanism is partly shared between Tph and Tfh cells in humans, which may be attributed to key cytokines: TGF-β and Activin A (8, 40, 41). Furthermore, IL-2/STAT5 pathway signaling inhibits the differentiation of Tfh and Tph cells (8, 40, 41).

Despite these similarities, there are some differences between Tph and Tfh cells (**Figure 2**). Tph cells do not express CXCR5, which is necessary for migration into GCs (37, 38). Therefore, Tph cells are supposed to be present in the periphery of TLSs, rather than in their center. Tph cells express low levels of Bcl6 while higher levels of the counter-regulator Blimp-1, a transcription factor typically downregulated in Tfh cells (7, 42, 43). In addition, Sox4 was identified as a key transcription factor for CXCL13 production by Tph cells, and increased Sox4 in CD4^+^ T cells was associated with TLS formation in RA synovium (44). Tph cells showed lower expression of CCR7 and CD27, but higher expression of CD44 and T-bet compared with Tfh cells, which might suggest a distinct migratory capacity (7, 45, 46). Flow cytometry revealed that Tph cells expressed high levels of inflammatory chemokine receptors, such as CCR2, CX3CR1, and CCR5, which might contribute to recruitment to inflammatory sites (7, 47). Although Tph cells are abundant in the inflamed tissues, PD-1^hi^CXCR5^−^CD4^+^ T cells can also be found in the circulation. Tissue Tph and circulating Tph (cTph) cells are cytometrically and transcriptomically similar populations, including increased expression of MHC II and ICOS, although the frequency of cTph cells is much lower (7). Functionally, both isolated cTph and circulating Tfh (cTfh) cells from patients with active established RA could provide efficient help to memory B cells, contributing to the production of IgG, IgA, and IgM. Nevertheless, when cTph cells were cocultured with naïve B cells, only IgM but not IgG or IgA was produced. In contrast, when cTfh cells were cocultured with naïve B cells, class-switched IgG and IgA were produced (48). The above results suggest that only cTfh rather than cTph cells are functionally equivalent to bona fide Tfh cells, while the function of cTph cells to help naïve B cells is limited. cTfh cells are thought to originate mainly from pre-Tfh cells in draining lymph nodes, while the origin of cTph cells is currently unknown (49). Interestingly, Tph cells may interact with extra-follicular “atypical memory” CD11c^+^CD21^−^ CXCR5^−^ B cells (50, 51).

## 4 Tph cells in human autoimmune diseases

Mounting studies have been conducted to investigate the physiological role and mechanisms of Tph cells, predominantly focusing on autoimmune diseases (shown in **Table 1**), including RA (6, 7, 48, 52–54), SLE (26, 55–61), SS (62–66), IgG4-related disease (IgG4-RD) (64, 67), type 1 diabetes (T1D) (68, 69), primary biliary cirrhosis (PBC) (70), immunoglobulin A nephropathy (IgAN) (71), juvenile idiopathic arthritis (JIA) (72), autoimmune hepatitis (AIH) (73), dermatomyositis (DM) (74), celiac disease (CeD) (58), systemic sclerosis (SSc) (58), autoimmune bowel disease (IBD) (75), and psoriasis vulgaris (PV) (76). Notably, most studies elucidated altered frequencies of Tph cells and their correlation with disease activity, and they analyzed only cTph cells because of the relative difficulty in obtaining tissue samples. Unfortunately, the relationship between cTph and tissue Tph cells has not been fully investigated.

**Table 1:** Studies of Tph cells in patients with autoimmune diseases.

Disease	Ref	Year	Patient cohort	Molecular phenotype	Main findings
**RA**	(7)	2017	Synovial tissue (n = 10), synovial fluid (n = 9), and blood (n = 42) from seropositive RA patients.	PD-1^hi^ CXCR5^−^ CD4^+^	Tph↑, PD-1^hi^MHC II^+^CXCR5^−^↑, and PD-1^hi^CXCR5^−^ICOS^+^↑ in seropositive RA patients. And these cells decreased in patients whose disease activity decreased after treatment escalation.
	(52)	2019	Synovial tissues from leukocyte-rich RA (n = 19) or leukocyte-poor RA (n = 17).	CD4^+^ PD-1^+^ ICOS^+^	CXCL13↑ in leukocyte-rich RA.
	(6)	2013	Synovial tissue cells from 2 patients and synovial fluid mononuclear cells from 8 patients.	CD4^+^ PD-1^+^ CXCR5^−^	Synovial Tph cells expressed CXCL13. TCR induced CXCL13 production, and proinflammatory cytokines supported the long-term production of CXCL13.
	(48)	2019	Fifty-six DMARD-naïve early RA patients including seropositive RA (n = 38) and seronegative RA (n = 18).	CD4^+^ CXCR5^−^ PD-1^hi^	cTph↑, and they were decreased with clinical improvement.
	(53)	2021	Thirty-four seropositive RA patients, and 11 seronegative RA patients.	PD-1^hi^ CXCR5^−^ CD4^+^	cTph↑, and HLA^−^DR^+^cTph↑ in seropositive RA patients. HLA^−^DR^−^ Tph cells reflected the disease activity.
	(54)	2020	Synovial samples from 11 patients on anti-TNF therapy.	PD-1^hi^ CXCR5^−^ CD4^+^	A lower abundance of synovial Tph cells was associated with a better response to anti-TNF therapy.
**SLE**	(55)	2019	Sixty-eight SLE patients including the active group (n = 22) and the inactive group (n = 46).	PD-1^+^ CXCR5^−^ CD4^+^	cTph↑, CD38^+^ cTph1↑, and Tph cells were correlated with lupus disease activity index and plasma cells.
	(56)	2019	Patients with SLE (n = 9).	CD3^+^ CD4^+^ CD45RA^−^ PD-1^high^ CXCR5^−^	cTph↑, cTph1↑, cTph were associated with lupus disease activity, and Tph1 cells were correlated with plasmablasts.
	(26)	2019	Lupus nephritis kidney biopsies (n = 13), lupus nephritis patients (n = 27), and PBMCs from SLE patients (n = 6).	PD-1^hi^ CXCR5^–^ CD4^+^	cTph↑, and they were correlated with disease activity and CD11c^+^ B cells. Tph promoted B cell responses in lupus *via* MAF and IL-21.
	(57)	2019	SLE patients displaying class II (n = 4), III (n = 9), class IV (n = 8) lupus nephritis.	CXCR5^−^ CXCR3^+^ PD1^hi^ CD4^+^	cTph↑, Tph↑ in the tubulointerstitial areas of patients with proliferative lupus nephritis. Tph cells provided B cell help through IL-10 and succinate.
	(58)	2019	N = 10 patients with SLE.	PD-1^+^ CXCR5^−^ CD4^+^	cTph↑ in 4/10 patients.
	(59)	2015	The blood of patients with SLE (n = 49).	CD4^+^ TCRβ^+^ CD45RA^−^ CXCR5^lo^	cTph↑, and they were correlated with disease activity and plasmablasts.
	(60)	2022	PBMCs from 52 SLE patients.	CD4^+^ PD-1^+^ CXCR5^−^	cTph↑, cTph/cTfh↑, and cTph cells were correlated with disease activity, serum levels of IFN-α, and renal involvement.
	(61)	2022	Ten lupus nephritis and 5 non-lupus nephritis SLE patients.	CD4^+^ PD-1^+^ CXCR5^−^	cTph and cTfh did not change after rituximab.
**SS**	(62)	2020	Blood and labial salivary glands biopsies from patients with SS (n = 83) and SS parotids with low-grade MALT-L (n = 15).	CD4^+^CD25^+^ CXCR5^−^ CD4^+^PD1^hi^ ICOS^+^ Foxp3^−^	cTph↑, SG Tph↑, and they frequently coexpressed IL-21/IFN-γ, especially in parotid MALT-L.
	(63)	2017	Patients with early and active primary SS (n = 15).	CD4^+^ CXCR5^−^ PD-1^high^	cTph↑, and they were decreased after abatacept treatment.
	(64)	2018	Blood specimens from patients with SS (n = 16).	PD-1^+^ CXCR5^−^ CD4^+^	The percentage of cTph↑, but the absolute number of cTph-.
	(65)	2022	Blood specimens (n = 60) and labial gland tissue biopsy specimens (n = 10) from patients with SS.	PD-1^+^ CXCR5^–^ CD4^+^	cTph↑, Tph↑ in labial gland, and cTph cells were correlated with disease activity and plasmablasts.
	(66)	2021	Blood specimens from 2 SS cohorts (n = 29 and n = 15).	CD4^+^ CXCR5^–^ PD-1^hi^	cTph↑, cTfh↑, but there was no correlation between ESSDAI and cTph or activated cTph cells. cTph cells were correlated with circulating plasmablasts.
**IgG4-RD**	(64)	2018	Blood specimens from patients with IgG4-RD (n = 53).	PD-1^+^ CXCR5^−^ CD4^+^	cTph↑, GZMA^+^cTph↑, cTph cells were positively correlated with serum IgG4, ratio of IgG4/IgG, number of organs involved, and sIL-2R.
	(67)	2021	Patients with IgG4-RD (n = 17).	PD-1^+^ CXCR5^−^ CD4^+^	CX3CR1^+^ cTph↑, and they were positively correlated with IgG4-RD responder index, number of organs involved, and serum level of sIL-2R.
**T1D**	(68)	2020	Abatacept administration, n = 34 patients.	CXCR5^−^ ICOS^+^ PD-1^+^	cTph↑ in individuals with new-onset T1D, and they were strongly reduced after abatacept treatment.
	(69)	2019	Forty-four children with newly diagnosed T1D.	CXCR5^−^ PD-1^hi^ CD4^+^	cTph↑, especially in those who are seropositive for multiple autoantibodies. cTph↑ in autoantibody-positive at-risk children who later progressed to T1D.
**PBC**	(70)	2021	Twenty PBC patients.	CD4^+^ CXCR5^−^ PD-1^+^	cTph↑, ICOS^+^ cTph↑, CD28^+^ cTph↑, and ICOS^+^ cTph cells were positively correlated with AMA-M2, IgM, and plasma cell levels.
**IgAN**	(71)	2020	Patients with IgAN (n = 37).	CD3^+^CD4^+^ PD1^hi^ CXCR5^−^	cTph↑, and they were correlated with disease severity. cTph cells reduced after treatment.
**JIA**	(72)	2022	Fifty-three patients with active JIA, in whom joint puncture had been performed for intraarticular steroid injection.	PD-1^high^ CXCR5^–^ HLA^–^DR^+^ CD4^+^	SF Tph cells differentiate B cell toward a CD21^low/–^CD11c^+^ phenotype *in vitro*, and frequencies of SF Tph cells were correlated with the appearance of SF CD21^low/–^CD11c^+^CD27^–^IgM^–^ double-negative B cells in situ.
**AIH**	(73)	2020	Patients with AIH with (SLA-pos; n = 8) and without (SLA-neg; n = 8) anti-SLA autoantibodies.	PD-1^+^ CXCR5^−^ CD4^+^	Memory Tph↑, CD45RA^−^PD-1^+^CD38^+^CXCR5^−^CD127^−^CD27^+^ subset was correlated with AIH activity.
**DM**	(74)	2021	Twenty-six newly diagnosed DM patients, and 15 patients were reanalyzed in remission during follow-up.	PD-1^hi^ CXCR5^−^ CD4^+^	cTph↓, cTfh↓, muscular Tph↑, and cTph↑ after treatment. cTph cells were negatively correlated with inflammation levels.
**CeD**	(58)	2019	Patients with untreated CeD (n = 8).	PD-1^+^ CXCR5^−^ CD4^+^	cTph↑ in 7/8 patients.
**SSc**	(58)	2019	Patients with SSc (n = 10).	PD-1^+^ CXCR5^−^ CD4^+^	cTph↑ in 8/10 patients.
**IBD**	(75)	2019	CD flare (n = 13); CD remission (n = 11); UC flare (n = 10); UC remission (n = 10).	CD3^+^CD4^+^CD45RO^+^ CXCR5^−^ PD-1^+^	Tissue Tph > cTph, Tph abundance in CD = that in UC.
**PV**	(76)	2021	Peripheral blood samples from 27 patients with PV.	CXCR3^−^ CCR6^+^ PD-1^+^ CXCR5^−^ CD4^+^	cTph17 cells had an activated, proliferative phenotype, and the quantity of cTph17 cells were positively correlated with disease severity, plasma CXCL13 levels, and cTfh cells.

Frequencies of Tph and Tfh cells were compared; ↓, lower level compared with controls; ↑, higher level compared with controls; -, no statistically significant difference between patients and controls; >, more than; =, no statistically significant difference between two groups.

Tph, T peripheral helper; ref, reference; RA, rheumatoid arthritis; PD-1, programmed cell death protein 1; CXCR, C‐X‐C chemokine receptor; MHC, major histocompatibility complex; ICOS, inducible T cell co-stimulator; CXCL13, C‐X‐C motif chemokine ligand 13; TCR, T-cell receptor; DMARD, disease-modify anti-rheumatic drug; cTph, circulating T peripheral helper; HLA, human leucocyte antigen; TNF, tumor-necrosis factor; SLE, systemic lupus erythematosus; PBMCs, peripheral blood mononuclear cells; IL, interleukin; IFN, interferon; cTfh, circulating follicular helper T; SS, sjögren syndrome; MALT-L, mucosa-associated lymphoid tissue lymphomas; SG, salivary gland; cTfh, circulating follicular helper T; ESSDAI, EULAR Sjogren’s syndrome disease activity index; IgG, immunoglobulin G; IgG4-RD, IgG4-related disease; GZMA, granzyme A; sIL-2R, soluble IL-2 receptor; T1D, type 1 diabetes; PBC, primary biliary cholangitis; AMA-M2, anti-mitochondrial antibodies against M2 antigen; IgAN, immunoglobulin A nephropathy; JIA, juvenile idiopathic arthritis; SF, synovial fluid; AIH, autoimmune hepatitis; SLA, anti-soluble liver antigen; DM, dermatomyositis; CeD, celiac disease; SSc, systemic sclerosis; IBD, autoimmune bowel disease; CD, Crohn’s disease; UC, ulcerative colitis; PV, psoriasis vulgaris.

RA is a chronic disease in which a person’s immune system attacks the synovium, causing pain, swelling, and stiffness (77). Tph cells were increased in patients with seropositive RA (7, 48, 53), while they were much lower in patients with seronegative RA and psoriatic arthritis (7). A decrease in Tph cell frequency was correlated with lower disease activity (7, 48). Tph cells did not vary with clinical indexes such as age, gender, disease duration, therapies, or serum levels of anti-citrullinated protein antibodies (7). HLA^−^DR^+^ Tph cells, HLA^−^DR^−^ Tph cells, and Tph1 cells were correlated with DAS28-ESR (53). HLA^−^DR^+^ Tph cells were decreased after methotrexate (MTX) treatment, independently of disease activity. However, HLA^−^DR^−^ Tph cells were correlated with DAS28-ESR during MTX treatment (53). In addition, a lower abundance of synovial Tph cells was associated with a better response to anti-TNF therapy (54). When RA tissue samples were further classified into leukocyte-poor RA and leukocyte-rich RA based on the cellular composition in synovial tissue, CXCL13, a chemokine expressed by Tph cells, was upregulated in bulk-sorted T cells from leukocyte-rich RA compared with that from osteoarthritis. The expression of marker genes of Tph cells was upregulated, indicating a higher abundance of Tph cells in leukocyte-rich RA than in osteoarthritis (52). Due to the production of CXCL13, Tph cells were also deemed as inflammatory CXCL13-producing helper T (iTh13) cells. T cell receptor induced CXCL13 production, and proinflammatory cytokines (IL-6 and TNF-α) supported the long-term production of CXCL13. Synovial Tph cells recruited CXCR5^+^ naive B cells and CXCR5^+^Tfh cells in a CXCL13-dependent manner (6). Tph cells could promote B cell differentiation into plasmablasts through the interactions between IL-21 and SLAMF5 (7).

SLE is an autoimmune connective-tissue disorder which is characterized by loss of self-tolerance and formation of nuclear autoantigens and immune complexes with great clinical heterogeneity (78). Tph cells were found to be enlarged in peripheral blood from SLE patients (26, 55–57, 59, 60) and in tubulointerstitial areas from patients with proliferative lupus nephritis (57). Four out of ten patients with SLE showed significantly elevated numbers of cTph cells (58). The frequency of Tph cells was correlated with lupus disease activity (26, 55, 56, 59, 60), plasma cells (55, 59), the frequency of CD11c^+^ B cells (26), serum levels of IFN-α (60), and renal involvement (60) in SLE patients. Th1 type Tph (Tph1) cells were expanded in patients with SLE (55, 56). Furthermore, the levels of MHC-II, ICOS, CD38, and IL-21 were upregulated in Tph cells from SLE patients (55). Mechanistically, Tph cells promoted B-cell responses in lupus through MAF, IL-21 (26), and through IL-10 and succinate (independent of IL-21) (57). In addition, IFN-α was shown to be essential for the development of Tph cells in SLE (60). IFN-α facilitated the expression of PD-1, IL-10, and Maf in TCR-activated CD4^+^T cells. Co-stimulation of IFN-α and IL-2 could induce the conversion of Tfh to Tph cells. Meanwhile, IFN-α-induced Tph cells could induce B cell differentiation into plasmablasts (60). In contrast, one study found no significant alteration in Tph cells after treatment with rituximab, suggesting that Tph cells do not change in response to disease activity (61).

SS is a systemic autoimmune disease which is characterized by lymphocytic infiltration of exocrine glands, mainly salivary and lacrimal glands (79). Tfh and Tph cells were enriched in both peripheral blood and salivary glands from SS patients, exhibiting significantly increased double IL-21/IFN-γ production but poor IL-17 expression, especially in parotid B-cell mucosa-associated lymphoid tissue-lymphoma. Furthermore, blockade by ICOS in ex vivo organ cultures significantly downregulated the production of IL-21, IL-6, IL-8, and TNF-α (62). cTph cells were significantly increased in patients with primary SS than in healthy controls (63, 65, 66). The proportions of Tph cells were decreased in patients treated with abatacept, and the numbers and proportions of cTph cells reverted to baseline levels once the treatment was stopped (63). As with cTfh cells, cTph cells were significantly correlated with CD138^+^/CD19^+^ plasma cells (65, 66) and disease activity parameters including EULAR Sjogren’s syndrome disease activity index (ESSDAI) scores, IgG, ESR, IL-21, and anti-SSA antibody (65). In contrast, another study found no correlation between ESSDAI and cTph or activated cTph cells (66). Furthermore, Tph cells were found in the labial gland tissue from patients with primary SS (65). Interestingly, one study found that the percentage of cTph cells was significantly increased in patients with SS than in healthy volunteers, whereas the absolute number of cTph cells was comparable to that in healthy volunteers (64).

IgG4-RD is an immunological disease presenting with abundant IgG4-positive plasma cells in affected tissues and fibrosis (80). The percentage and absolute number of Tph cells were increased in IgG4-RD patients compared with those in healthy volunteers. Furthermore, the percentage of Tph cells was positively correlated with clinical parameters of IgG4-RD, including serum levels of IgG4, ratio of IgG4/IgG, number of organs involved, and soluble IL-2 receptor (sIL-2R). Interestingly, granzyme A^+^ cells were abundantly enriched in Tph cells, and they were significantly elevated in IgG4-RD patients. Also, the percentage and absolute number of Tph cells were decreased after treatment with glucocorticoids (64). The study team further found that CX3CR1 was highly expressed in Tph cells from IgG4-RD patients. The percentage of CX3CR1^+^ Tph cells was positively correlated with clinical parameters including IgG4-RD responder index, number of organs involved, and serum level of sIL-2R, but not with IgG and IgG4. Granzyme A, perforin, and G protein-coupled receptor 56 were highly expressed in CX3CR1^+^ Tph cells and they were cytotoxic to vascular endothelial cells and ductal epithelial cells (67).

T1D is a T cell-mediated autoimmune disease characterized by β-cell destruction and dysfunction, which is preceded by a period of asymptomatic autoimmunity possessing multiple islet autoantibodies (81, 82). The frequency of cTph cells was increased in children with newly diagnosed T1D, especially in those who were seropositive for multiple autoantibodies. Furthermore, cTph cells were increased in autoantibody-positive at-risk children who later progressed to T1D. These results revealed the association of cTph cells with progression to T1D, and therefore cTph cells are suggested to be considered as a biomarker to predict disease progression and as a potential target for immunotherapy (69). cTph cells were found to be increased in patients with new-onset T1D, and they were strongly reduced after abatacept treatment at both year 1 and year 2 (68).

PBC is a chronic autoimmune cholestatic liver disease, which is characterized by slow and progressive destruction of small intrahepatic bile ducts, contributing to fibrosis, potential cirrhosis and related complications (83). The frequencies of cTph cells, ICOS^+^ cTph cells, and CD28^+^ cTph cells were increased in patients with PBC. The levels of ICOS^+^ cTph cells were upregulated in PBC patients than in healthy controls, and they were downregulated after treatment. Furthermore, the levels of ICOS^+^ cTph cells were positively correlated with anti-mitochondrial antibodies against M2 antigen, IgM, and plasma cell levels. Therefore, it is suggested that the activation status of cTph cells is related to the severity of PBC (70).

IgAN is the most prevalent primary glomerular disease worldwide and is a dominant cause of renal failure in East Asian countries (84). The frequencies of various cTph cell subsets were significantly higher in patients with IgAN compared with those in healthy controls, and they were negatively correlated with estimated glomerular filtration rate before treatment. The percentage of cTph cells was positively correlated with the percentage of CD138^+^ B cells. The percentage of different subsets of circulating PD-1^hi^CXCR5^−^ T cells, CD138^+^ B cells, and serum IL-21 concentration were significantly reduced after corticosteroid treatment (71).

JIA is the most common chronic inflammatory rheumatic condition of childhood that comprises seven categories of arthritis of unknown origin (85). Tph cells were found to be accumulated in the joints of ANA-positive JIA patients. SF Tph cells induced plasma cell differentiation, Ig secretion, and skewed B cell differentiation toward a CD21^low/–^CD11c^+^ phenotype *in vitro* by providing IL-21 and IFN-γ. In addition, frequencies of SF Tph cells were correlated with the appearance of SF CD21^low/–^CD11c^+^CD27^–^IgM^–^ double-negative B cells in situ. Clonally expanded Tph cells appeared to represent a pathogenic T cell subset by promoting CD21^low/–^CD21^low/–^CD11c^+^ double-negative B cell differentiation (72).

AIH is a severe liver disease characterized by elevated serum transaminase and immunoglobulin G levels, the presence of autoantibodies, and interface hepatitis on liver histology (86). Memory PD-1^+^CXCR5^−^ CD4^+^ T cells with a Tph profile were found to be enriched in the blood of patients with AIH. Soluble liver antigen-specific CD4^+^ T cells had a transcriptomic signature resembling the Tph signature. A specific T-cell phenotypic signature was further identified, namely CD45RA^−^PD-1^+^CD38^+^CXCR5^−^CD127^−^CD27^+^, which supported B cell differentiation *via* producing IL-21 correlated with AIH activity (including transaminase and serum IgG levels). Thus, it is speculated that this subset could be used to track and/or target pathogenic T cells in AIH (73).

DM is a subgroup of idiopathic inflammatory myopathies, which is defined by the coexistence of characteristic myositis and cutaneous manifestations (87). Decreased cTph and cTfh cells were found in DM patients than in healthy controls. In contrast, the level of Tph cells in muscle was increased, and the accumulated B cells located around Tph cells in infiltrated lesions (74). The frequency of cTph cells was positively correlated with Tfh and CD3^+^/CD4^+^/CD8^+^ T cells, whereas negatively correlated with inflammation levels such as erythrocyte sedimentation rate, IL-6, and IL-10. Furthermore, the decreased cTph cells were upregulated after glucocorticoid treatment (74).

CeD is an autoimmune enteropathy against dietary gluten that occurs in genetically predisposed individuals (88). Except for CXCR5, which was not detected, a subset of cells with upregulated markers including CD38, CD39, CXCR3, PD-1, ICOS, CD161, CCR5, CD28, CD200, CD84, CXCL13, and IL-21 was demonstrated. Compared with controls, seven out of eight patients with untreated CeD showed significantly elevated numbers of CD4^+^ T cells with this phenotype in peripheral blood mononuclear cells (58).

SSc is a complex autoimmune disease characterized by fibrosis of the skin and visceral organs (89). Compared with controls, upregulated Tph cells were found in the circulation in eight out of ten patients with SSc (58).

IBD, including Crohn’s disease (CD) and ulcerative colitis (UC), is a chronic relapsing/remitting immune-mediated disease which is triggered by environmental factors, genetics, and gut microbiota (90). No differences in Tph cell abundance were found between CD and UC patients. Nevertheless, in both diseases, the frequency of Tph cells was significantly higher in tissues than in blood. Despite no difference in Tph abundance between CD and UC tissues, Tph cells had significantly higher pSTAT3 expression in CD tissues than in UC tissues (75).

PV is a chronic non-infectious disease that influences the skin, nails, and joints and is associated with multiple comorbidities (91). An activated, proliferative phenotype of cTph17 cells was found in PV, and the quantity of cTph17 cells was positively correlated with disease severity. Plasma CXCL13 levels were elevated and associated with the frequency of Tph17 cells and disease severity. CD4^+^CXCR5^+^ cTfh cells were increased in patients and positively correlated with disease severity, frequency of Tph17 cells, and plasma CXCL13 levels (76). Therefore, cTph17 cells and CXCL13/CXCR5 axis may represent new immunotherapeutic targets for PV.

## 5 Conclusion and perspectives

Accumulating evidence has highlighted the importance of Tph cells in autoimmune diseases. Considering the alterations of Tph cells in the above-mentioned diseases, Tph cells have several potential applications. Tph cells may be a novel disease biomarker and therapeutic target for autoimmune diseases. In addition, they could predict clinical response to immunotherapy or even disease progression, as in T1D. However, we must be aware of the divergencies among these findings. Depending on the inflammatory signals from different tissues, the characteristics of Tph cells may differ in diseases. Patients with different stages of the disease, therapeutic regimens, and different gating approaches may account for part of the reason. The different phenotypes and functions of Tph cells may be determined by the current stage of the immune response and their different localization in tissues, as in the case of Tfh and Tfr cells (49). The biology of Tph cells is still poorly understood, including their origin, differentiation and mechanisms of Tph-cell effector functions. Single-cell technology should be used to further dissect the heterogeneity within Tph cells. Future research should also emphasize in-depth mechanisms and functions rather than just numerical alterations in diseases. Undoubtedly, bona fide Tph cells in tissue samples should be put to wider use. Further clarification of the molecular mechanisms of Tph cells and the crosstalk between T and B cells would contribute to a better understanding of the pathogenesis of autoimmune diseases and the development of immunotherapies.

## Author contributions

YH wrote the first draft of the manuscript. All authors contributed to the article and approved the submitted version.

## References

[1] Szekanecz Z McInnes IB Schett G Szamosi S Benko S Szucs G. Autoinflammation and autoimmunity across rheumatic and musculoskeletal diseases. Nat Rev Rheumatol (2021) 17:585–95. doi: 10.1038/s41584-021-00652-9 34341562

[2] Huang Y Chen Z Wang H Ba X Shen P Lin W. Follicular regulatory T cells: a novel target for immunotherapy? Clin Transl Immunol (2020) 9:e1106. doi: 10.1002/cti2.1106 PMC7019198 32082569

[3] Crotty S. T Follicular helper cell biology: A decade of discovery and diseases. Immunity (2019) 50:1132–48. doi: 10.1016/j.immuni.2019.04.011 PMC6532429 31117010

[4] Manzo A Vitolo B Humby F Caporali R Jarrossay D Dell’accio F. Mature antigen-experienced T helper cells synthesize and secrete the b cell chemoattractant CXCL13 in the inflammatory environment of the rheumatoid joint. Arthritis Rheum (2008) 58:3377–87. doi: 10.1002/art.23966 18975336

[5] Pitzalis C Jones GW Bombardieri M Jones SA. Ectopic lymphoid-like structures in infection. Cancer autoimmunity Nat Rev Immunol (2014) 14:447–62. doi: 10.1038/nri3700 24948366

[6] Kobayashi S Murata K Shibuya H Morita M Ishikawa M Furu M. A distinct human CD4+ T cell subset that secretes CXCL13 in rheumatoid synovium. Arthritis Rheum (2013) 65:3063–72. doi: 10.1002/art.38173 24022618

[7] Rao DA Gurish MF Marshall JL Slowikowski K Fonseka CY Liu Y. Pathologically expanded peripheral T helper cell subset drives b cells in rheumatoid arthritis. Nature (2017) 542:110–4. doi: 10.1038/nature20810 PMC5349321 28150777

[8] Yoshitomi H Ueno H. Shared and distinct roles of T peripheral helper and T follicular helper cells in human diseases. Cell Mol Immunol (2021) 18:523–7. doi: 10.1038/s41423-020-00529-z PMC8027819 32868910

[9] Yoshitomi H. Peripheral helper T cell responses in human diseases. Front Immunol (2022) 13:946786. doi: 10.3389/fimmu.2022.946786 35880181 PMC9307902

[10] Gago da Graca C van Baarsen LGM Mebius RE. Tertiary lymphoid structures: Diversity in their development, composition, and role. J Immunol (2021) 206:273–81. doi: 10.4049/jimmunol.2000873 33397741

[11] Antonioli L Fornai M Pellegrini C Masi S Puxeddu I Blandizzi C. Ectopic lymphoid organs and immune-mediated diseases: Molecular basis for pharmacological approaches. Trends Mol Med (2020) 26:1021–33. doi: 10.1016/j.molmed.2020.06.004 32600794

[12] Schumacher TN Thommen DS. Tertiary lymphoid structures in cancer. Science (2022) 375:eabf9419. doi: 10.1126/science.abf9419 34990248

[13] Shi K Hayashida K Kaneko M Hashimoto J Tomita T Lipsky PE. Lymphoid chemokine b cell-attracting chemokine-1 (CXCL13) is expressed in germinal center of ectopic lymphoid follicles within the synovium of chronic arthritis patients. J Immunol (2001) 166:650–5. doi: 10.4049/jimmunol.166.1.650 11123349

[14] Hansen A Lipsky PE Dorner T. B cells in sjogren’s syndrome: indications for disturbed selection and differentiation in ectopic lymphoid tissue. Arthritis Res Ther (2007) 9:218. doi: 10.1186/ar2210 17697366 PMC2206371

[15] Golub R Cumano A. Embryonic hematopoiesis. Blood Cells Mol Dis (2013) 51:226–31. doi: 10.1016/j.bcmd.2013.08.004 24041595

[16] Pitzalis C Kelly S Humby F. New learnings on the pathophysiology of RA from synovial biopsies. Curr Opin Rheumatol (2013) 25:334–44. doi: 10.1097/BOR.0b013e32835fd8eb 23492740

[17] Humby F Bombardieri M Manzo A Kelly S Blades MC Kirkham B. Ectopic lymphoid structures support ongoing production of class-switched autoantibodies in rheumatoid synovium. PloS Med (2009) 6:e1. doi: 10.1371/journal.pmed.0060001 PMC2621263 19143467

[18] Manzo A Paoletti S Carulli M Blades MC Barone F Yanni G. Systematic microanatomical analysis of CXCL13 and CCL21 *in situ* production and progressive lymphoid organization in rheumatoid synovitis. Eur J Immunol (2005) 35:1347–59. doi: 10.1002/eji.200425830 15832291

[19] Takemura S Braun A Crowson C Kurtin PJ Cofield RH O’Fallon WM. Lymphoid neogenesis in rheumatoid synovitis. J Immunol (2001) 167:1072–80. doi: 10.4049/jimmunol.167.2.1072 11441118

[20] Thurlings RM Vos K Wijbrandts CA Zwinderman AH Gerlag DM Tak PP. Synovial tissue response to rituximab: mechanism of action and identification of biomarkers of response. Ann Rheum Dis (2008) 67:917–25. doi: 10.1136/ard.2007.080960 PMC2564787 17965121

[21] Thurlings RM Wijbrandts CA Mebius RE Cantaert T Dinant HJ van der Pouw-Kraan TC. Synovial lymphoid neogenesis does not define a specific clinical rheumatoid arthritis phenotype. Arthritis Rheum (2008) 58:1582–9. doi: 10.1002/art.23505 18512774

[22] Barone F Gardner DH Nayar S Steinthal N Buckley CD Luther SA. Stromal fibroblasts in tertiary lymphoid structures: A novel target in chronic inflammation. Front Immunol (2016) 7:477. doi: 10.3389/fimmu.2016.00477 27877173 PMC5100680

[23] Buckley CD Barone F Nayar S Benezech C Caamano J. Stromal cells in chronic inflammation and tertiary lymphoid organ formation. Annu Rev Immunol (2015) 33:715–45. doi: 10.1146/annurev-immunol-032713-120252 25861980

[24] Pipi E Nayar S Gardner DH Colafrancesco S Smith C Barone F. Tertiary lymphoid structures: Autoimmunity goes local. Front Immunol (2018) 9:1952. doi: 10.3389/fimmu.2018.01952 30258435 PMC6143705

[25] Marks KE Rao DA. T Peripheral helper cells in autoimmune diseases. Immunol Rev (2022) 307:191–202. doi: 10.1111/imr.13069 35103314 PMC9009135

[26] Bocharnikov AV Keegan J Wacleche VS Cao Y Fonseka CY Wang G. Accelerating medicines partnership, PD-1hiCXCR5- T peripheral helper cells promote b cell responses in lupus *via* MAF and IL-21. JCI Insight (2019) 4. doi: 10.1172/jci.insight.130062 PMC6824311 31536480

[27] Fonseca VR Ribeiro F Graca L. T Follicular regulatory (Tfr) cells: Dissecting the complexity of tfr-cell compartments. Immunol Rev (2019) 288:112–27. doi: 10.1111/imr.12739 30874344

[28] Noel G Fontsa ML Garaud S De Silva P de Wind A Van den Eynden GG. Functional Th1-oriented T follicular helper cells that infiltrate human breast cancer promote effective adaptive immunity. J Clin Invest (2021) 131. doi: 10.1172/JCI139905 PMC8483751 34411002

[29] Chang A Henderson SG Brandt D Liu N Guttikonda R Hsieh C. *In situ* b cell-mediated immune responses and tubulointerstitial inflammation in human lupus nephritis. J Immunol (2011) 186:1849–60. doi: 10.4049/jimmunol.1001983 PMC3124090 21187439

[30] Arahata K Engel AG. Monoclonal antibody analysis of mononuclear cells in myopathies. I: Quantitation of subsets according to diagnosis and sites of accumulation and demonstration and counts of muscle fibers invaded by T cells. Ann Neurol (1984) 16:193–208. doi: 10.1002/ana.410160206 6383191

[31] De Bleecker JL Engel AG Butcher EC. Peripheral lymphoid tissue-like adhesion molecule expression in nodular infiltrates in inflammatory myopathies. Neuromuscul Disord (1996) 6:255–60. doi: 10.1016/0960-8966(96)00015-6 8887954

[32] Muramatsu M Kinoshita K Fagarasan S Yamada S Shinkai Y Honjo T. Class switch recombination and hypermutation require activation-induced cytidine deaminase (AID), a potential RNA editing enzyme. Cell (2000) 102:553–63. doi: 10.1016/S0092-8674(00)00078-7 11007474

[33] Corsiero E Bombardieri M Carlotti E Pratesi F Robinson W Migliorini P. Single cell cloning and recombinant monoclonal antibodies generation from RA synovial b cells reveal frequent targeting of citrullinated histones of NETs. Ann Rheum Dis (2016) 75:1866–75. doi: 10.1136/annrheumdis-2015-208356 PMC5036240 26659717

[34] Bombardieri M Lewis M Pitzalis C. Ectopic lymphoid neogenesis in rheumatic autoimmune diseases. Nat Rev Rheumatol (2017) 13:141–54. doi: 10.1038/nrrheum.2016.217 28202919

[35] Bauer L Muller LJ Volkers SM Heinrich F Mashreghi MF Ruppert C. Follicular helper-like T cells in the lung highlight a novel role of b cells in sarcoidosis. Am J Respir Crit Care Med (2021) 204:1403–17. doi: 10.1164/rccm.202012-4423OC PMC8865704 34534436

[36] Hutloff A. T Follicular helper-like cells in inflamed non-lymphoid tissues. Front Immunol (2018) 9:1707. doi: 10.3389/fimmu.2018.01707 30083164 PMC6064731

[37] Schaerli P Willimann K Lang AB Lipp M Loetscher P Moser B. CXC chemokine receptor 5 expression defines follicular homing T cells with b cell helper function. J Exp Med (2000) 192:1553–62. doi: 10.1084/jem.192.11.1553 PMC2193097 11104798

[38] Breitfeld D Ohl L Kremmer E Ellwart J Sallusto F Lipp M. Follicular b helper T cells express CXC chemokine receptor 5, localize to b cell follicles, and support immunoglobulin production. J Exp Med (2000) 192:1545–52. doi: 10.1084/jem.192.11.1545 PMC2193094 11104797

[39] Cannons JL Qi H Lu KT Dutta M Gomez-Rodriguez J Cheng J. Optimal germinal center responses require a multistage T cell:B cell adhesion process involving integrins, SLAM-associated protein, and CD84. Immunity (2010) 32:253–65. doi: 10.1016/j.immuni.2010.01.010 PMC2830297 20153220

[40] Schmitt N Liu Y Bentebibel SE Munagala I Bourdery L Venuprasad K. The cytokine TGF-beta co-opts signaling *via* STAT3-STAT4 to promote the differentiation of human TFH cells. Nat Immunol (2014) 15:856–65. doi: 10.1038/ni.2947 PMC4183221 25064073

[41] Kobayashi S Watanabe T Suzuki R Furu M Ito H Ito J. TGF-beta induces the differentiation of human CXCL13-producing CD4(+) T cells. Eur J Immunol (2016) 46:360–71. doi: 10.1002/eji.201546043 PMC5063156 26541894

[42] Johnston RJ Poholek AC DiToro D Yusuf I Eto D Barnett B. Bcl6 and blimp-1 are reciprocal and antagonistic regulators of T follicular helper cell differentiation. Science (2009) 325:1006–10. doi: 10.1126/science.1175870 PMC2766560 19608860

[43] Crotty S. Follicular helper CD4 T cells (TFH). Annu Rev Immunol (2011) 29:621–63. doi: 10.1146/annurev-immunol-031210-101400 21314428

[44] Yoshitomi H Kobayashi S Miyagawa-Hayashino A Okahata A Doi K Nishitani K. Human Sox4 facilitates the development of CXCL13-producing helper T cells in inflammatory environments. Nat Commun (2018) 9:3762. doi: 10.1038/s41467-018-06187-0 30232328 PMC6145936

[45] DeGrendele HC Estess P Siegelman MH. Requirement for CD44 in activated T cell extravasation into an inflammatory site. Science (1997) 278:672–5. doi: 10.1126/science.278.5338.672 9381175

[46] Forster R Schubel A Breitfeld D Kremmer E Renner-Muller I Wolf E. CCR7 coordinates the primary immune response by establishing functional microenvironments in secondary lymphoid organs. Cell (1999) 99:23–33. doi: 10.1016/S0092-8674(00)80059-8 10520991

[47] Rot A von Andrian UH. Chemokines in innate and adaptive host defense: basic chemokinese grammar for immune cells. Annu Rev Immunol (2004) 22:891–928. doi: 10.1146/annurev.immunol.22.012703.104543 15032599

[48] Fortea-Gordo P Nuno L Villalba A Peiteado D Monjo I Sanchez-Mateos P. Two populations of circulating PD-1hiCD4 T cells with distinct b cell helping capacity are elevated in early rheumatoid arthritis. Rheumatology (2019) 58:1662–73. doi: 10.1093/rheumatology/kez169 31056653

[49] Deng J Wei Y Fonseca VR Graca L Yu D. T Follicular helper cells and T follicular regulatory cells in rheumatic diseases. Nat Rev Rheumatol (2019) 15:475–90. doi: 10.1038/s41584-019-0254-2 31289377

[50] Jenks SA Cashman KS Zumaquero E Marigorta UM Patel AV Wang X. Distinct effector b cells induced by unregulated toll-like receptor 7 contribute to pathogenic responses in systemic lupus erythematosus. Immunity (2018) 49:725–739.e6. doi: 10.1016/j.immuni.2018.08.015 30314758 PMC6217820

[51] Jenks SA Cashman KS Woodruff MC Lee FE Sanz I. Extrafollicular responses in humans and SLE. Immunol Rev (2019) 288:136–48. doi: 10.1111/imr.12741 PMC6422038 30874345

[52] Zhang F Wei K Slowikowski K Fonseka CY Rao DA Kelly S. Defining inflammatory cell states in rheumatoid arthritis joint synovial tissues by integrating single-cell transcriptomics and mass cytometry. Nat Immunol (2019) 20:928–42. doi: 10.1038/s41590-019-0378-1 PMC6602051 31061532

[53] Yamada H Sasaki T Matsumoto K Suzuki K Takeshita M Tanemura S. Distinct features between HLA-DR+ and HLA-DR- PD-1hi CXCR5- T peripheral helper cells in seropositive rheumatoid arthritis. Rheumatology (2021) 60:451–60. doi: 10.1093/rheumatology/keaa417 32885242

[54] Julia A Avila G Celis R Sanmarti R Ramirez J Marsal S. Lower peripheral helper T cell levels in the synovium are associated with a better response to anti-TNF therapy in rheumatoid arthritis. Arthritis Res Ther (2020) 22:196. doi: 10.1186/s13075-020-02287-9 32843099 PMC7446220

[55] Lin J Yu Y Ma J Ren C Chen W. PD-1+CXCR5-CD4+T cells are correlated with the severity of systemic lupus erythematosus. Rheumatology (2019) 58:2188–92. doi: 10.1093/rheumatology/kez228 31180450

[56] Makiyama A Chiba A Noto D Murayama G Yamaji K Tamura N. Expanded circulating peripheral helper T cells in systemic lupus erythematosus: association with disease activity and b cell differentiation. Rheumatology (2019) 58:1861–9. doi: 10.1093/rheumatology/kez077 30879065

[57] Caielli S Veiga DT Balasubramanian P Athale S Domic B Murat E. A CD4(+) T cell population expanded in lupus blood provides b cell help through interleukin-10 and succinate. Nat Med (2019) 25:75–81. doi: 10.1038/s41591-018-0254-9 30478422 PMC6325012

[58] Christophersen A Lund EG Snir O Sola E Kanduri C Dahal-Koirala S. Distinct phenotype of CD4(+) T cells driving celiac disease identified in multiple autoimmune conditions. Nat Med (2019) 25:734–7. doi: 10.1038/s41591-019-0403-9 PMC6647859 30911136

[59] Choi JY Ho JH Pasoto SG Bunin V Kim ST Carrasco S. Circulating follicular helper-like T cells in systemic lupus erythematosus: association with disease activity. Arthritis Rheumatol (2015) 67:988–99. doi: 10.1002/art.39020 PMC4450082 25581113

[60] Jiang Q Wang J Jiang H Li W Sun Y Shan Y. Competitive binding of transcription factors underlies flexibility of T peripheral helper cells and T follicular helper cells in SLE. Rheumatology (2022). 61(11):4547–57. doi: 10.1093/rheumatology/keac112 35191465

[61] Faustini F Sippl N Stalesen R Chemin K Dunn N Fogdell-Hahn A. Rituximab in systemic lupus erythematosus: Transient effects on autoimmunity associated lymphocyte phenotypes and implications for immunogenicity. Front Immunol (2022) 13:826152. doi: 10.3389/fimmu.2022.826152 35464461 PMC9027571

[62] Pontarini E Murray-Brown WJ Croia C Lucchesi D Conway J Rivellese F. Unique expansion of IL-21+ tfh and tph cells under control of ICOS identifies sjogren’s syndrome with ectopic germinal centres and MALT lymphoma. Ann Rheum Dis (2020) 79:1588–99. doi: 10.1136/annrheumdis-2020-217646 PMC7677495 32963045

[63] Verstappen GM Meiners PM Corneth OBJ Visser A Arends S Abdulahad WH. Attenuation of follicular helper T cell-dependent b cell hyperactivity by abatacept treatment in primary sjogren’s syndrome. Arthritis Rheumatol (2017) 69:1850–61. doi: 10.1002/art.40165 28564491

[64] Kamekura R Yamamoto M Takano K Yabe H Ito F Ikegami I. Circulating PD-1(+)CXCR5(-)CD4(+) T cells underlying the immunological mechanisms of IgG4-related disease. Rheumatol Adv Pract (2018) 2:rky043. doi: 10.1093/rap/rky043 31431980 PMC6649940

[65] Chen W Yang F Lin J. Tph cells expanded in primary sjogren’s syndrome. Front Med (2022) 9:900349. doi: 10.3389/fmed.2022.900349 PMC9218540 35755031

[66] Dupre A Pascaud J Riviere E Paoletti A Ly B Mingueneau M. Association between T follicular helper cells and T peripheral helper cells with b-cell biomarkers and disease activity in primary sjogren syndrome. RMD Open (2021) 7. doi: 10.1136/rmdopen-2020-001442 PMC7944988 33688082

[67] Yabe H Kamekura R Yamamoto M Murayama K Kamiya S Ikegami I. Cytotoxic tph-like cells are involved in persistent tissue damage in IgG4-related disease. Mod Rheumatol (2021) 31:249–60. doi: 10.1080/14397595.2020.1719576 32023137

[68] Edner NM Heuts F Thomas N Wang CJ Petersone L Kenefeck R. Follicular helper T cell profiles predict response to costimulation blockade in type 1 diabetes. Nat Immunol (2020) 21:1244–55. doi: 10.1038/s41590-020-0744-z PMC7610476 32747817

[69] Ekman I Ihantola EL Viisanen T Rao DA Nanto-Salonen K Knip M. Circulating CXCR5(-)PD-1(hi) peripheral T helper cells are associated with progression to type 1 diabetes. Diabetologia (2019) 62:1681–8. doi: 10.1007/s00125-019-4936-8 PMC6677711 31270583

[70] Yong L Chunyan W Yan Y Wanyu L Huifan J Pingwei Z. Expanded circulating peripheral helper T cells in primary biliary cholangitis: Tph cells in PBC. Mol Immunol (2021) 131:44–50. doi: 10.1016/j.molimm.2020.09.007 33446391

[71] Wang X Li T Si R Chen J Qu Z Jiang Y. Increased frequency of PD-1(hi)CXCR5(-) T cells and b cells in patients with newly diagnosed IgA nephropathy. Sci Rep (2020) 10:492. doi: 10.1038/s41598-019-57324-8 31949193 PMC6965632

[72] Fischer J Dirks J Klaussner J Haase G Holl-Wieden A Hofmann C. Effect of clonally expanded PD-1(high) CXCR5-CD4+ peripheral T helper cells on b cell differentiation in the joints of patients with antinuclear antibody-positive juvenile idiopathic arthritis. Arthritis Rheumatol (2022) 74:150–62. doi: 10.1002/art.41913 34196496

[73] Renand A Cervera-Marzal I Gil L Dong C Garcia A Kervagoret E. Integrative molecular profiling of autoreactive CD4 T cells in autoimmune hepatitis. J Hepatol (2020) 73:1379–90. doi: 10.1016/j.jhep.2020.05.053 32649971

[74] Hou X Yang C Lin M Tian B Zhao S Liu X. Altered peripheral helper T cells in peripheral blood and muscle tissue of the patients with dermatomyositis. Clin Exp Med (2021) 21:655–61. doi: 10.1007/s10238-021-00713-z 33900488

[75] Rubin SJS Bai L Haileselassie Y Garay G Yun C Becker L. Mass cytometry reveals systemic and local immune signatures that distinguish inflammatory bowel diseases. Nat Commun (2019) 10:2686. doi: 10.1038/s41467-019-10387-7 31217423 PMC6584653

[76] Liu W Zhou X Wang A Ma and Y. Bai J. Increased peripheral helper T cells type 17 subset correlates with the severity of psoriasis vulgaris. Immunol Lett (2021) 229:48–54. doi: 10.1016/j.imlet.2020.11.005 33232721

[77] Smith MH Berman JR. What is rheumatoid arthritis? JAMA (2022) 327:1194. doi: 10.1001/jama.2022.0786 35315883

[78] Durcan L O’Dwyer T Petri M. Management strategies and future directions for systemic lupus erythematosus in adults. Lancet (2019) 393:2332–43. doi: 10.1016/S0140-6736(19)30237-5 31180030

[79] Brito-Zeron P Baldini C Bootsma H Bowman SJ Jonsson R Mariette X. Sjogren syndrome. Nat Rev Dis Primers (2016) 2:16047. doi: 10.1038/nrdp.2016.47 27383445

[80] Lanzillotta M Mancuso G Della-Torre E. Advances in the diagnosis and management of IgG4 related disease. BMJ (2020) 369:m1067. doi: 10.1136/bmj.m1067 32546500

[81] Herold KC Vignali DA Cooke A Bluestone JA. Type 1 diabetes: translating mechanistic observations into effective clinical outcomes. Nat Rev Immunol (2013) 13:243–56. doi: 10.1038/nri3422 PMC4172461 23524461

[82] Ziegler AG Rewers M Simell O Simell T Lempainen J Steck A. Seroconversion to multiple islet autoantibodies and risk of progression to diabetes in children. JAMA (2013) 309:2473–9. doi: 10.1001/jama.2013.6285 PMC4878912 23780460

[83] Carey EJ Ali AH Lindor KD. Primary biliary cirrhosis. Lancet (2015) 386:1565–75. doi: 10.1016/S0140-6736(15)00154-3 26364546

[84] Pattrapornpisut P Avila-Casado C Reich HN. IgA nephropathy: Core curriculum 2021. Am J Kidney Dis (2021) 78:429–41. doi: 10.1053/j.ajkd.2021.01.024 34247883

[85] Martini A Lovell DJ Albani S Brunner HI Hyrich KL Thompson SD. Juvenile idiopathic arthritis. Nat Rev Dis Primers (2022) 8:5. doi: 10.1038/s41572-021-00332-8 35087087

[86] Mieli-Vergani G Vergani D Czaja AJ Manns MP Krawitt EL Vierling JM. Autoimmune hepatitis. Nat Rev Dis Primers (2018) 4:18017. doi: 10.1038/nrdp.2018.17 29644994

[87] Lundberg IE Fujimoto M Vencovsky J Aggarwal R Holmqvist M Christopher-Stine L. Idiopathic inflammatory myopathies. Nat Rev Dis Primers (2021) 7:86. doi: 10.1038/s41572-021-00321-x 34857798

[88] Lindfors K Ciacci C Kurppa K Lundin KEA Makharia GK Mearin ML. Coeliac disease. Nat Rev Dis Primers (2019) 5:3. doi: 10.1038/s41572-018-0054-z 30631077

[89] Allanore Y Simms R Distler O Trojanowska M Pope J Denton CP. Systemic sclerosis. Nat Rev Dis Primers (2015) 1:15002. doi: 10.1038/nrdp.2015.2 27189141

[90] Ananthakrishnan AN. Epidemiology and risk factors for IBD. Nat Rev Gastroenterol Hepatol (2015) 12:205–17. doi: 10.1038/nrgastro.2015.34 25732745

[91] Michalek IM Loring B John SM. A systematic review of worldwide epidemiology of psoriasis. J Eur Acad Dermatol Venereol (2017) 31:205–12. doi: 10.1111/jdv.13854 27573025

